# Effects of intrapolyp steroid injection on intraocular pressure and recurrent polyp treatment

**DOI:** 10.1038/s41433-021-01719-3

**Published:** 2021-09-16

**Authors:** Tae-Hoon Lee, Jung-Gwon Nam, Chang Kyu Lee

**Affiliations:** 1 Department of Otolaryngology-Head and Neck Surgery, Ulsan University Hospital, University of Ulsan College of Medicine, Ulsan, South Korea; 2 Department of Ophthalmology, Ulsan University Hospital, University of Ulsan College of Medicine, Ulsan, South Korea

**Keywords:** Glaucoma, Lacrimal apparatus diseases

## Abstract

**Purpose:**

To examine the effects of intrapolyp triamcinolone acetonide (TA) injections on intraocular pressure (IOP) and recurrence of nasal polyps after endoscopic sinus surgery.

**Patients and methods:**

This was a prospective, randomized, double-blind, placebo-controlled study. Patients were randomized into either the TA injection group (Group I, 20 mg/ml, 2 mL) or the placebo normal saline injection group (group II, 2 mL). There were a total of five study visits: one baseline visit and one at 2, 4, 8, and 12 w after the injection. The primary safe outcome was the change in IOP between two groups at 4 w. The secondary safe outcome was the IOP at each visit and proportion of patients having IOP above 21 mmHg at 4 and 8 w. Changes in the nasal polyp size were measured between two groups at each visit.

**Results:**

A total of 43 consenting participants completed this study (22 in group I and 21 in group II). The mean IOP elevation in both eyes was not significantly different between the groups (*p* > 0.05) and was not over 2 mmHg at the 4-w mark. There was also no significant difference in the proportion of patients having IOP above 21 mmHg at 4 and 8 w between the groups (*p* > 0.05). However, there was a significant difference in the change in polyp size until 8 w between both groups (*p* < 0.01).

**Conclusions:**

Intrapolyp TA injection is a safe and effective method for the management of recurrent polyps after endoscopic sinus surgery.

## Introduction

The primary treatment methods for nasal polyps after endoscopic sinus surgery (ESS) are medical treatment with steroids, biologics, and surgery. Steroid treatments can be administered either topically or orally. Oral steroids are usually prescribed short-term, for up to 2 w, owing to the risk of systemic side effects such as Cushing’s syndrome because of endocrine disruption. Systemic steroids, such oral steroids, can increase the intraocular pressure (IOP), which may lead to the development of cataracts or glaucoma following long-term use [1]. In contrast, intranasal corticosteroid sprays (INCS) are targeted to the nose and have minimal systemic absorption, allowing them to be used safely without the risk of side effects. Although caution is necessary in patients with glaucoma and cataracts, no significant relationship has been reported between IOP elevation and development of cataract in healthy individuals [2].

Conversely, intravitreal triamcinolone acetonide (TA) injection, used for the treatment of uveitis or vitreoretinal disease, can cause IOP elevation by increasing the extracellular matrix of the trabecular meshwork, leading to an accumulation of glycosaminoglycan and preventing intraocular fluid leakage. The IOP elevation is more with TA than with than other steroids because of its high potency and long duration of use [3–5].

Intrapolyp steroid injections are effective as a treatment method as high concentrations of steroids act directly and locally on the nasal polyps and surrounding nasal mucosa [6]. In addition, it has a long effect (~6–8 weeks) without systemic side effects, and therefore can be used as an alternative to oral steroids [7].

Unlike INCSs, oral corticosteroids, and intravitreal steroid injections, intrapolyp steroid injections have not previously been studied as a treatment method for recurrent nasal polyps after ESS or regarding its ocular side effects. Since the nasal cavity and the orbit is in contact, intrapolyp steroid injections can cause side effects such as IOP elevation and cataracts. The purpose of this study was to evaluate the effects of intrapolyp TA injection on IOP, glaucoma onset, and recurrent nasal polyps after ESS.

## Subjects and methods

### Subjects

This study adhered to the tenets of the Declaration of Helsinki, and approval was obtained from the Ulsan University Institutional Review Board (UUH 2017-08-004). All the patients were enrolled between November 2017 and February 2019. This study was designed to be prospective, double-blind, and randomized and was registered at the Clinical Research Information Service (KCT0003127).

Patients over the age of 19 y who underwent ESS and in whom nasal polyps recurred during the follow-up period were enrolled in this study. Informed consent was obtained from all the patients. Patients who had glaucoma, a high baseline IOP (≥21 mmHg), myopia (axial length >26 mm or spherical equivalent < −6D), connective tissue disorder, and/or diabetes were excluded from this study because they were more vulnerable to steroid therapy [8–12]. Pregnant or lactating women were also excluded.

### Technique

Before intrapolyp injection, the TA was diluted to 2 mL of a 40 mg/mL solution (Dongkwang Pharmaceutical Co., LTD., Seoul, South Korea). Vasoconstriction was then induced using phenylephrine-soaked nasal gauzes. Using a 1-mL tuberculin syringe and a 26-gauge spinal needle for each nasal cavity, the intrapolyp injection was administered under 30-degree endoscope visualization. The spinal needle tip was frequently bent to inject the polyps in the frontoethmoidal area. We injected 1 mL of diluted TA (intervention group) or normal saline (control group) into each nasal cavity, and another 2 mL of diluted TA (40 mg) or normal saline into the entire nasal cavity.

### Data collection

A randomized schedule, which was prepared and sealed in advance, was used to divide the patients into two groups: the TA group (TA injection, 40 mg) and the normal saline group. The intrapolyp injection was administered by two otolaryngologists (T.H.L. and J.G.N.), and the IOP was measured by one glaucoma specialist (C.K.L.). All the patients were followed at 2, 4, 8, and 12 w post-injection. We attempted to measure the IOPs in both eyes at the same time in the morning to rule out diurnal IOP variations during the day. The IOPs were measured three times per visit using Goldmann applanation tonometry, and the mean values were used as representative values [13–15]. The same glaucoma specialist conducted the measurements; however, no information about the injection materials was provided during the follow-up period.

We assessed the safety of intrapolyp TA injection by measuring the changes in IOP. The primary safety outcome was the difference between the groups in terms of the change in IOP at 4 w, i.e., 4 w–0 w IOP (ΔIOP-4 w), which meant comparing the IOP at 4 w to that at the baseline. We decided to measure the IOP at 4 w because most previous studies that assessed TA injection and IOP showed the highest elevation 4 w after the injection [16–18]. For the secondary safety outcomes, the IOPs at all follow-up points and the proportion of patients over 21 mmHg at 4 and 8 w in the groups were compared. Finally, we assessed the glaucomatous change at the final visit (12 w). If glaucomatous change was noted, we considered that a severe adverse event and were prepared to start glaucoma treatment. The glaucoma diagnosis criteria were as follows: (1) a cup-to-disc ratio ≥0.5 or a >0.2 difference in the cup-to-disc ratio of each eye; (2) a defect in the retinal nerve fiber layer (consistent with a glaucomatous change in the optic nerve) on either a fundus photograph taken using red-free light or an optical coherence tomography image; and (3) evidence of glaucomatous visual field loss using a Humphrey Field Analyzer (with the Swedish Interactive Threshold Algorithm-Standard 30-2 or 24-2 program).

During the follow-up period, if a patient withdrew from the study or if the IOP was above 26 mmHg, we planned to analyse the glaucomatous change regardless of the time post-injection. We also considered this a severe adverse event.

Changes in nasal polyp size were determined using endoscopy at the time of injection and at 2, 4, 8, and 12 w post-injection. The nasal polyp size was scored as follows: 0, absent; 1, limited to the middle meatus; 2, extending to the nasal cavity.

### Data analysis

The IOP change and primary safety outcome (ΔIOP-4 w) were analysed using the paired *t* test. The proportion of patients with an IOP above 21 mmHg at 4 and 8 w was analysed using Fisher’s exact test. The polyp size change was analysed using the independent *t* test.

The study population was calculated to verify the difference in IOP at 4 w post-operation (ΔIOP-4 w) between the study and control groups. This study was designed as an equivalence study. The upper limit was set at 2 mmHg based on the normal ocular pressure range of 15 ± 2.5 mmHg [19], and the intra-individual variation of the Goldman tonometer was set under 3 mmHg [20, 21]. One-sided alpha level was set at 0.05, the power, 80%, and the dropout rate, 20%. Finally, the study population of each group was calculated as 25.

Statistical significance was determined based on *P* < 0.05 and was analysed using IBM SPSS Statistics ver. 24 (IBM Corp., Armonk, NY, USA).

## Results

### Demographic characteristics

**Fig. 1:**
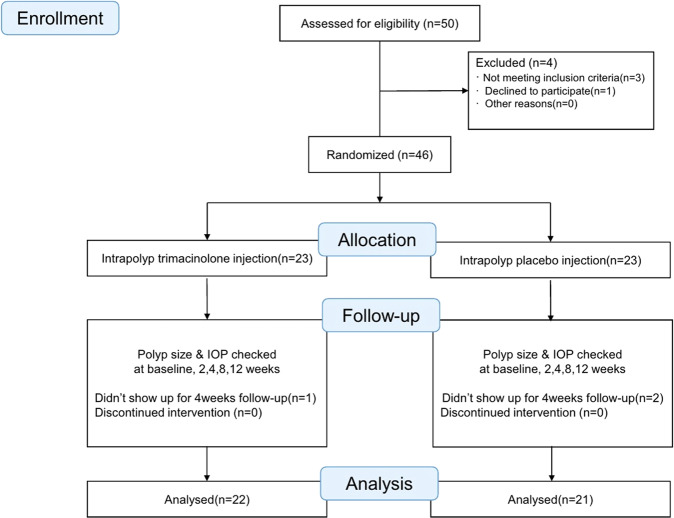
Patients Enrollment. Flow diagram of the study design and patient inclusion criteria.

In total, 50 cases were assessed for study eligibility; four cases were excluded. The remaining 46 cases were randomly assigned at a 1:1 ratio to receive either the intrapolyp TA injection (group I) or the placebo intrapolyp normal saline injection (group II) at the beginning of the study. Three cases were lost during the study follow-up period. A final total of 43 cases were included in this study (Fig. 1). There were no significant differences in sex or age between the two groups (Table 1).

**Table 1:** Baseline patient characteristics.

	Group I (*n* = 22)	Group II (*n* = 21)	*P* value
Age, year (mean ± SD)	51.41 ± 14.98	47.79 ± 12.96	0.25
Sex, men/women	13/9	14/7	0.94
HTN (%)	42.8	25.5	0.16

Group I: trimacinolone acetonide injection group, Group II: placebo (normal saline) injection group.

*SD* standard deviation.

### Outcome

The primary safety outcome measured was the increase in IOP at 4 w. In this study, there was no significant difference in IOP increase in both eyes between both groups at 4 w (Table 2).

**Table 2:** Difference in the increase of IOP between the groups at four weeks.

		Group I (*n* = 22)	Group II (*n* = 21)	*P* value
ΔIOP-4 wk (mean)	Rt. Eye	−0.382	0.535	0.11
	Lt. Eye	−0.500	0.005	0.31

ΔIOP-4wk: difference in IOP from baseline to 4 weeks; Group I: trimacinolone acetonide injection group; Group II: placebo (normal saline) injection group;

statistical analysis method: Paired *t* test.

**Fig. 2:**
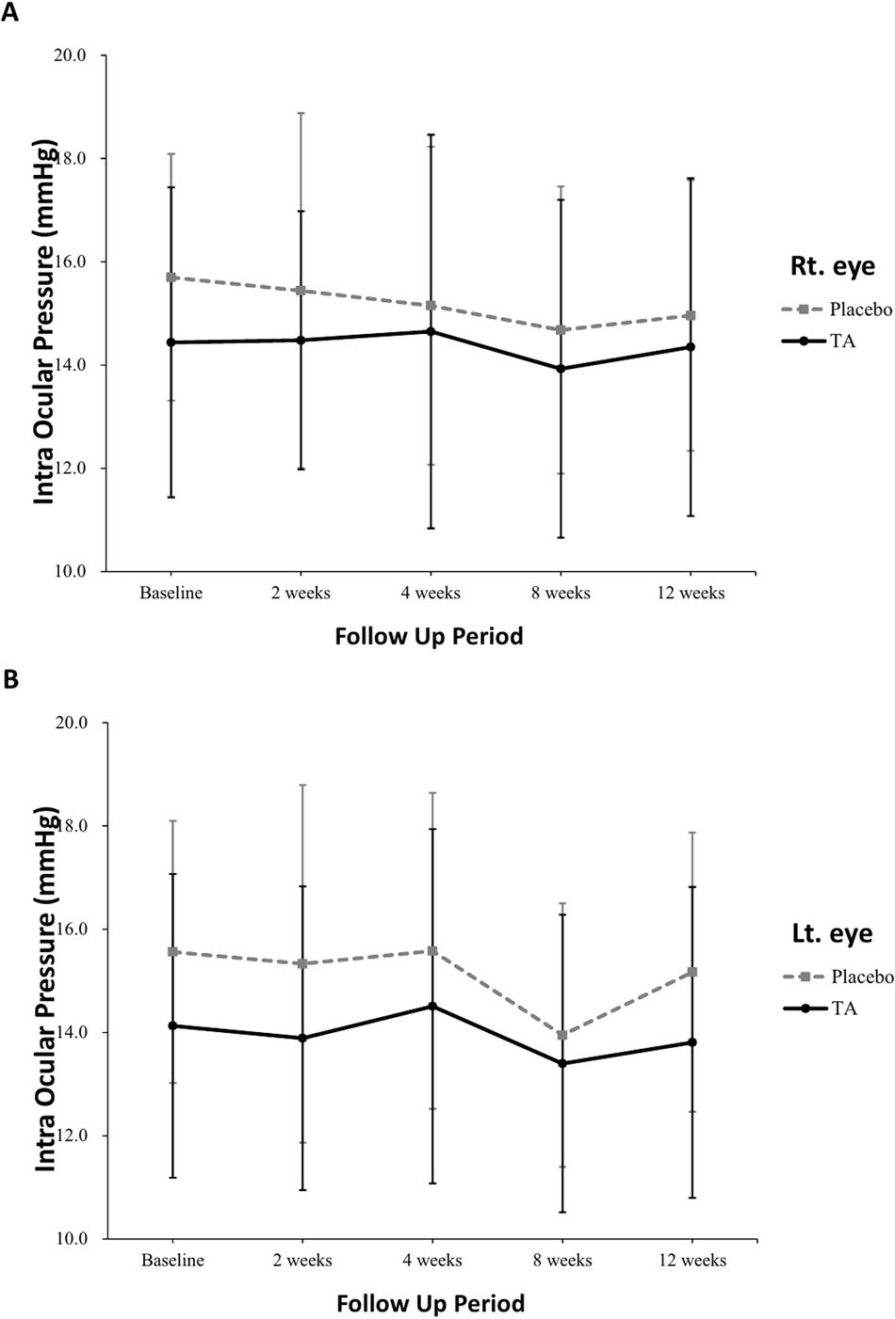
Graph comparing the intraocular pressure between both groups at each follow-up period. There was no significant difference between both groups. (**A** = Right eye, **B** = Left eye).

The secondary outcomes measured compared the groups’ IOPs at all follow-up points and compared the proportion of participants with IOPs ≥21 mmHg at 4 and 8 w. There was no point where the IOPs in both eyes of group I were significantly higher than those of group II during the follow-up period (Fig. 2).

There was also no difference between the groups in terms of the proportion of participants with IOPs ≥21 mmHg at 4 w. However, at 8 w, the proportion of participants with >21 mmHg in group I was higher than that in group II, though the difference was not significant (Table 3).

**Table 3:** Proportion of participants with IOP over 21 mmHg at 4 and 8 weeks in both groups.

		Group I *N* (%)	Group II *N* (%)	*P* value
4 weeks	Rt. eye	2 (9.1)	1 (5)	1.00
	Lt. eye	1 (4.5)	2 (10)	0.59
8 weeks	Rt. eye	1 (4.5)	0 (0.0)	1.00
	Lt. eye	1 (4.5)	0 (0.0)	1.00

Group I: trimacinolone acetonide injection group; Group II: placebo (normal saline) injection group; statistical analysis method: Fisher’s exact test.

No patient was diagnosed with glaucoma at the last follow-up point or was excluded due to high IOP (>26 mmHg) during the entire follow-up period.

There was a significant difference in the change in polyp size between the two groups until 8 w. However, at 12 w, the difference was not significant (Table 4).

**Table 4:** Changes in nasal polyps during follow-up in both groups.

	Group I	Group II	*P* value
0 weeks	1.00 ± 0.00	1.00 ± 0.00	–
2 weeks	0.67 ± 0.48	0.93 ± 0.26	0.008
4 weeks	0.42 ± 0.50	0.83 ± 0.38	0.001
8 weeks	0.24 ± 0.44	0.66 ± 0.48	0.001
12 weeks	0.27 ± 0.45	0.48 ± 0.51	0.093

Group I: trimacinolone acetonide injection group; Group II: placebo (normal saline) injection group; statistical analysis method: Independent *t* test.

### Complications

There were no major complications, such as visual loss or visual field defects, and no minor complications, such as bleeding or infection.

## Discussion

Intranasal steroid injections were first introduced in the 1950s as a treatment for allergic rhinitis and nasal polyposis and has shown excellent therapeutic results [22, 23]. However, in the 1960s, cases of visual loss were reported, most of which occurred when the solution was injected into the turbinate or the septum [24, 25]. Injected steroid particles may cause retrograde embolization in the central retinal artery through the anterior or posterior ethmoid artery and then the ophthalmic artery. In order to prevent this, a guideline has been suggested: first, selection of a small particle steroid, such as TA; second, using a thin needle smaller than a 25-gauge spinal needle; and third, injection after topical vasoconstriction [26]. Since these guidelines were proposed in the 1980s, the occurrence of permanent visual loss has been rarely reported.

Although INCSs are routinely used to prevent the recurrence of nasal polyps after ESS, it is often inadequate. Oral corticosteroids are effective in the treatment of recurrent nasal polyps, but their effect is temporary and the risk of systemic side effects increases as the dose increases [27]. Kiris et al. reported that intrapolyp steroid injections used in the treatment of nasal polyps showed subjective and objective effects similar to that of short-term oral corticosteroids, with normal blood cortisol and adrenocorticotropic hormone levels and no major side effects [6]. In this study of patients with recurrent nasal polyps after surgery, we demonstrated similar results. In addition to INCSs after surgery, intrapolyp steroid injections may be a good alternative to oral corticosteroids for the treatment of recurrent nasal polyps. This may be especially useful in high-risk patients for oral corticosteroids, such as those with diabetes. Intrapolyp TA injections can be very effective in the treatment of recurrent nasal polyps in the ethmoid sinus and nasofrontal duct, but treatment of a recurrent nasal polyp in the maxillary sinus is limited because the needle cannot reach this area.

Intravitreal TA (IVTA; 2–4 mg) is used for the treatment of pseudophakic macular oedema, uveitis, choroidal neovascularization, macular oedema following diabetic retinopathy, and central retinal vein occlusion [28–33]. Reported complications of IVTA injections include cataract formation, endophthalmitis, and elevated IOP, which is the most common complication [28, 34–37]. Elevated IOP is an important, treatable risk factor for the development of glaucomatous optic neuropathy.

The precise mechanism of a steroid–induced increase in IOP could be downregulation of trabecular matrix metalloproteinase activity, increased myocilin production, or decreased trabecular phagocytic activity [37–39]. These biochemical events result in an increased resistance to aqueous outflow at the level of the trabecular meshwork and may be triggered by steroids administered via any route.

According to Barteselli et al., patients who received a 20-mg injection of IVTA who did not regularly use anti-glaucoma drugs post-injection had an IOP elevation of 7 mmHg by 1 mon, with a maximum IOP ≥21 mmHg seen in 52% of the study population [17]. However, using the standard TA dose of 4 mg, mild to moderate IOP elevation has been reported in 18.5% of patients, typically within the first 3 mon following injection [16, 34]. Aref’s study also showed that the rate of IOP-related events was higher in the 4-mg IVTA group compared that in to the 1-mg IVTA group [16]. These results showed that complications of TA injection, such as IOP elevation and cataract development, were strongly related to the dose of TA. This means that a high dose of TA (40 mg) may result in more IOP elevation events than a standard dose (4 mg), which is 10 times the dose of TA in our study. Therefore, we hypothesized that a high intrapolyp injection dose of TA (40 mg) may lead to IOP elevation after injection, even though the injection site is not the orbit but the nasal cavity nearest the space of orbit. However, our study showed that there were no IOP elevation events related to TA nasal polyp injections.

We considered several reasons for these findings. The first possible reason was related to the pharmacokinetic characteristics of TA. Important factors in the relationship between IOP elevation and TA are not only systemic but also local bioavailability. The systemic bioavailability of TA was confirmed by the relationship between the amount of TA and the frequency of TA complications. However, the systemic bioavailability of intranasal TA has been shown to be only 46%. This also correlates with the low lipophilicity of TA [40]. Increased lipophilicity correlates with a greater binding affinity for and prolonged occupation of the corticosteroid receptor and, consequently, less unbound drug to interact with systemic glucocorticoid receptors, which can potentially result in adverse events [41]. Moreover, nasal polyps have low vascularity. Therefore, the systemic bioavailability of TA after intranasal polyp injection may be lower than that after intranasal mucosal TA injection. Moreover, the local bioavailability of TA is also an important factor in the elevation of IOP. A study by Hirano et al. compared IOP elevation after IVTA injection to that after posterior sub-Tenon capsule TA injection (STTA). They found that the IOP elevation was significantly slower in STTA compared to that in IVTA, and that cases of high IOP and IOP elevation in STTA were lower than that in IVTA [18]. This may mean that the ocular space, which is the closest area of trabecular meshwork during TA injection, is important for the elevation of IOP. Moreover, the orbit is separated from the sinus cavity by surrounding bones, such as the frontal bone, lacrimal bone, zygoma, maxilla, ethmoid, sphenoid, and palate [42]. Therefore, intranasal TA injection is only affected by its systemic bioavailability, which is lower than that of dexamethasone and flunisolide, with no effect on local bioavailability [41].

The one of this study’s limitations was generalizability because of the small number of cases, even though we had already examined the study population. And another limitation of this study was that patients at high risk for glaucoma development were excluded from the study. Patients with diabetes, preexisting glaucoma, high baseline IOP, high myopia, cardiovascular diseases, and connective tissue diseases are most likely at risk for glaucomatous changes and represent the population that both ophthalmologists and otolaryngologists may be most concerned about when starting intrapolyp TA injections. Therefore, although we found no significant differences between the TA injection group and the placebo group, our study highlights the need to formally assess ocular changes in high-risk patient populations receiving intrapolyp TA injections to identify if our findings were consistent in that population.

Our ophthalmology assessment findings demonstrate that high doses of TA injection can be safely used for polyps or ostium granulomas after endoscopic dacryocystorhinostomy in healthy patients. Jo et al. previously reported that an intralesional TA injection (12 mg) can help regress ostium granulomas and considered that steroids had definite effects on inflammatory granulomas [43]. Therefore, in such cases, ophthalmologists can use high doses of TA injection on granulomas to safely increase the surgical success rates.

In conclusion, high-dose intrapolyp TA injections can be safely used in ophthalmological procedures with respect to the IOP and is an effective treatment for recurrent nasal polyp after ESS.

### Summary

#### What was known before

- High doses of intravitreal steroid injection (triamcinolone acetonide) can increase intraocular pressure.
- Therefore, clinicians should pay attention after intravitreal steroid injection.

#### What this study adds

- High doses of intranasal polyp triamcinolone acetonide (40 mg) injection did not increase intraocular pressure and decrease polyp size.
- Therefore, for clinicians who want to decrease polyp size after DCR or ESS surgery with safety, it could be good treatment option.
